# Improvement in Sitting Balance by Exercises With a Standing Unstable Board Combined With Transcutaneous Electrical Nerve Stimulation to the Neck: A Study of Two Cases

**DOI:** 10.7759/cureus.93047

**Published:** 2025-09-23

**Authors:** Koki Nagai, Kazu Amimoto, Yumi Ikeda

**Affiliations:** 1 Department of Rehabilitation, Medical Corporation Sonodakai, Hanahata Rehabilitation Hospital, Tokyo, JPN; 2 Department of Physical Therapy, Tokyo Metropolitan University, Graduate School of Health Sciences, Tokyo, JPN; 3 Department of Physical Therapy, Sendai Seiyo Gakuin University, Sendai, JPN

**Keywords:** lateral sitting balance, neck muscle stimulation, righting reaction, stroke, transcutaneous electrical nerve stimulation, unstable board

## Abstract

Unstable board training and neck muscle stimulation have each been shown to improve sitting balance in stroke patients, but their combined effects remain unclear. This case report investigated whether transcutaneous electrical nerve stimulation (TENS) applied to the neck enhances the effectiveness of unstable board training on lateral sitting balance in individuals with severe subacute stroke. Two stroke survivors who required substantial assistance with activities of daily living participated in an alternating AB design intervention. In period A, both patients received conventional physiotherapy and unstable board training; in period B, neck TENS was included to the same program. Each physical therapy session lasted 40 minutes, consisting of 20 minutes of conventional physiotherapy and 20 minutes of unstable board training. During unstable board training, the non-paralyzed lower limb was placed on an unstable board, whereas the paralyzed limb, stabilized with a knee-ankle-foot orthosis, was placed on a stable surface. Patients performed weight-shifting and multidirectional reaching tasks under supervision. In period B, TENS was applied to the paralyzed sternocleidomastoid muscle at 100 Hz and 200 µs pulse width for 20 minutes during unstable board training. Righting reaction angles and center of pressure displacement were measured in the sitting position. Both outcomes improved to a greater extent in period B than in period A, particularly in the patient with milder sensory and motor impairments. These findings suggest that TENS applied to the neck may enhance sensory integration and postural adaptation when combined with unstable board training. The greater effect observed in the case with preserved somatosensory function implies that individual patient characteristics may influence the effectiveness of this combined intervention. This approach may offer a safe and potentially effective method to improve sitting balance in stroke patients with severe motor deficits. Further studies with larger samples and neurophysiological assessments are needed to confirm these preliminary observations.

## Introduction

Sitting balance in patients with acute or subacute stroke is strongly associated with trunk motor control [1,2], a key determinant of independence in activities of daily living (ADL) [3]. Among various components of balance, lateral sitting balance is particularly predictive of functional outcomes, necessitating early and targeted intervention [4]. Existing trunk function assessments [5,6] are established methods for assessing trunk function and sitting balance in stroke patients. However, these assessments are interval-scale and may lack sensitivity to subtle changes. Furthermore, severe hemiplegia increases the risk of falls, highlighting the need for a safe and accurate assessment of lateral sitting balance. Considering this, we developed a method to quantify righting reaction (RR), which has shown high reliability [7]. The RR angle and center of pressure (COP) displacement during lateral tilts using a vertical board (VB) have been established as reliable measures, even in patients with severe stroke [7,8]. These assessments provide insight into compensatory strategies and trunk function [8,9]. Although the direct validity of the RR angle as an outcome measure has not yet been fully established, indirect evidence supports its clinical relevance: improvements in the RR angle have been reported following unstable board training [8], which targets trunk function. Patients with milder hemiplegia, who also showed higher Trunk Impairment Scale (TIS) scores, could achieve RR primarily with the trunk rather than through compensatory neck or non-paralyzed leg movements [10]. Taken together, these findings suggest that the RR angle is closely related to trunk motor control and compensatory strategies, indirectly supporting its construct validity. In this study, RR does not refer to a traditionally "reflexive" phenomenon, but instead to a "voluntary" action of repositioning oneself upright against gravity from a laterally tilted sitting position; this action is defined as behavioral verticality and trunk control [10].

Unstable board training is known to enhance dynamic postural control by facilitating proprioceptive feedback and sensorimotor integration [11,12]. Neuroimaging studies have suggested that it may promote plasticity in brain areas involved in postural adaptation [13]. However, owing to fall risk, its use has often been limited to patients with mild impairment. To address this, we previously developed a modified method for severely affected individuals, in which the paralyzed leg is stabilized with a knee-ankle-foot orthosis (KAFO) and the board is placed under the non-paralyzed leg [8]. This approach improved the trunk RR angle and COP displacement; however, the degree of improvement may vary among individuals, and further enhancement strategies have not been explored yet.

Stimulation of the neck muscles, including transcutaneous electrical nerve stimulation (TENS) and vibratory stimulation, activates the sternocleidomastoid muscle and related vestibular pathways, thereby contributing to improved balance by activating the insular cortex and somatosensory areas [14,15]. TENS can also improve postural control in stroke patients with spatial neglect [16]. Unstable board training and neck muscle stimulation act through different mechanisms of sensory input and postural regulation; therefore, combining these methods may provide complementary effects and lead to quicker and more consistent improvements in trunk function and sitting balance. However, the efficacy of this combined approach remains unclear.

Taken together, unstable board training and neck TENS each offer benefits through distinct mechanisms, but either approach alone may be insufficient for patients with severe stroke. Their combination has the potential to produce complementary effects, simultaneously enhancing proprioceptive, vestibular, and somatosensory input, and may therefore yield greater or more consistent improvements in sitting balance. Evaluating the efficacy of this combined approach is important for identifying clinically applicable strategies to optimize rehabilitation outcomes.

This study aimed to investigate whether adding neck TENS enhances the efficacy of unstable board training in improving sitting balance in patients with severe stroke. We hypothesized that TENS would enhance sensory input and promote sensorimotor integration, leading to greater postural improvement through multisensory stimulation. These findings align with established neurorehabilitation principles of motor learning and brain plasticity [17].

## Case presentation

Case introduction

**Figure 1:**
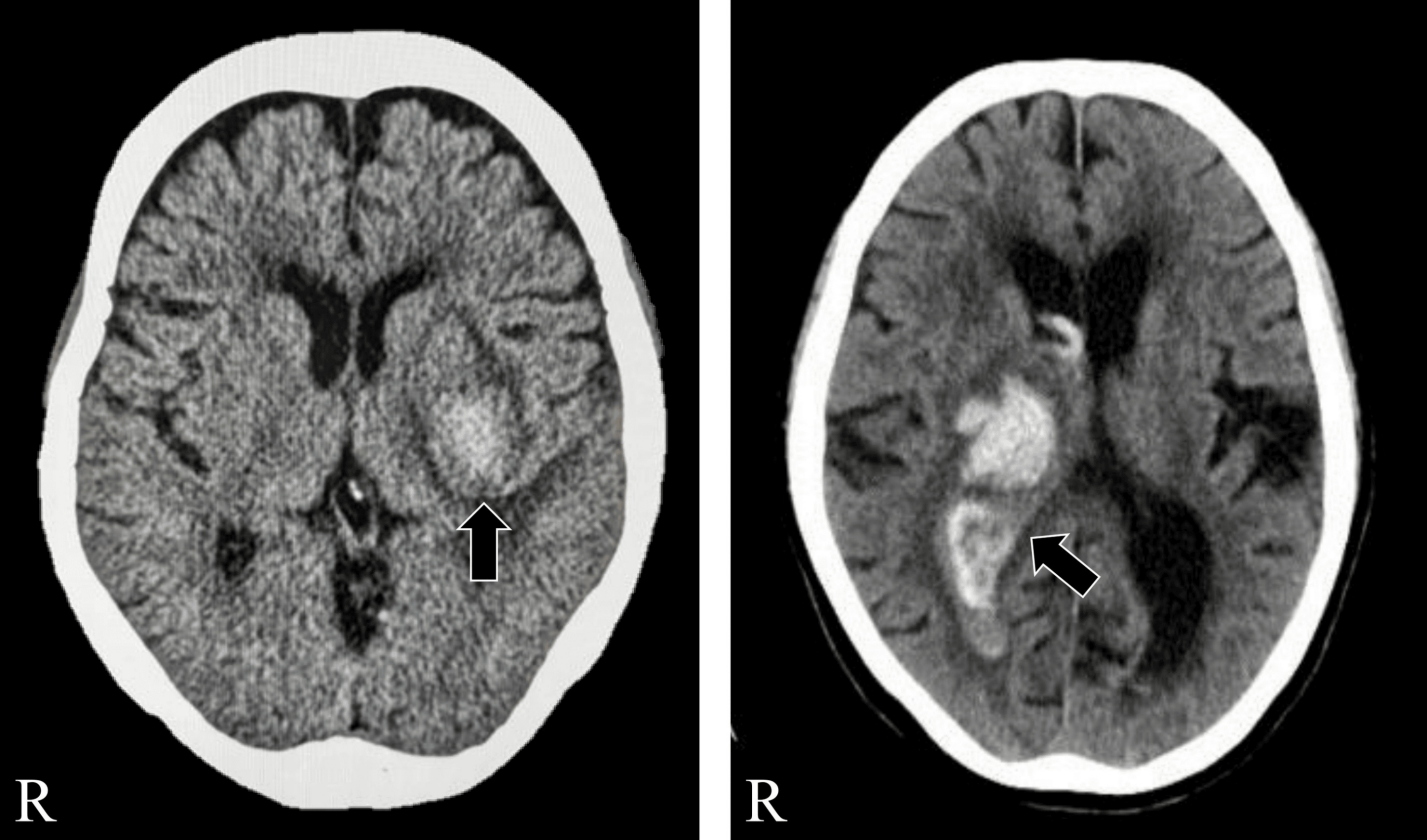
Computed tomography of the patients. Computed tomography of case 1 on the left and case 2 on the right.

The clinical characteristics of case 1 and case 2 are summarized in Table 1, with corresponding computed tomography (CT) images shown in Figure 1. Based on previous research [8], participants were selected from patients with hemiplegia who had a modified Ranking Scale score of 5 and required severe or total assistance with ADLs but could maintain a sitting position for at least one minute. Among those who met these criteria, we present two cases that differed in sensory function, time since onset, and baseline trunk control to illustrate how these factors may influence responsiveness to the combined intervention.

**Table 1:** Neurological and neuropsychological evaluations. BRS, Brunnstrom Recovery Stage; FMA, Fugl-Meyer Assessment; FAC, Functional Ambulation Categories; MMSE, Mini-Mental State Examination. ^a^: BRS, upper extremity/finger/lower extremity. ^b^: FMA, upper extremity/lower extremity. ^c^: Maximum score of 30 points (cutoff: 23 points).

		Case 1	Case 2
Age (years)		74	75
Sex		Female	Female
Days from the onset (days)		62	116
Diagnosis		Hemorrhage	Hemorrhage
Lesion area		Left putamen	Right thalamus
BRS^a^		Ⅱ/Ⅱ/Ⅱ	Ⅳ/Ⅳ/Ⅳ
FMA^b^ (points)		55/60	54/77
FAC (points)		1	0
Sensory test	Superficial sensory	Sever	Mild
	Deep sensory	Mild	Mild
MMSE^c^ (points)		26	23

Case 1

This was the case of a 74-year-old woman (height: 162 cm, weight: 43 kg, body mass index (BMI): 16.2 kg/m²) with right hemiplegia due to left putaminal hemorrhage. CT revealed compression of the insular cortex, internal capsule, and thalamus, with preservation of the primary somatosensory cortex. Her Fugl-Meyer Assessment (FMA) scores were 55 (upper limb) and 60 (lower limb). The Brunnstrom Recovery Stage (BRS) was II for the upper limb, fingers, and lower limb. She had severe superficial and mild deep sensory impairment but no aphasia. These sensory impairments were observed to a similar extent in both the paralyzed upper and lower limbs. The Functional Ambulation Categories (FAC) score was 1. Her TIS [5] and Mini-Mental State Examination (MMSE) scores were 11 and 26, respectively. Her Functional Independence Measure (FIM) scores were 32 (motor), 11 (cognitive), and 43 (total). The evaluation was conducted 116 days post stroke.

Case 2

This was the case of a 75-year-old woman (height: 152 cm, weight: 42 kg, BMI: 22.5 kg/m²) with left hemiplegia due to right thalamic hemorrhage. CT showed compression of the internal capsule, with intact primary somatosensory and insular cortices. Her FMA scores were 54 (upper limb) and 77 (lower limb), and her BRS was IV across all limbs. She presented with mild unilateral spatial neglect but no pusher behavior. During her hospitalization, she participated in rehabilitation sessions and actively practiced toileting with the assistance of nursing staff. The FAC score was 0. Sensory impairment was mild for both superficial and deep modalities. These deficits were also comparable in the paralyzed upper and lower limbs. Her TIS and MMSE scores were 4 and 23, respectively, and her FIM scores were 26 (motor), 14 (cognitive), and 40 (total). The evaluation was conducted 62 days post stroke.

Ethical considerations

This study was conducted in accordance with the Declaration of Helsinki and approved by the Ethics Committee of Sonodakai (approval number: 122) and the Research Ethics Committee of Tokyo Metropolitan University Arakawa Campus (approval number: 22054). Written and verbal consent was obtained from all participants before they participated in the study.

Intervention design

The intervention used an AB alternative design. Each intervention period lasted one week, and evaluations were conducted at the beginning and end of each period. The total intervention duration was two weeks. Participants received 40 minutes of physical therapy, occupational therapy, and speech therapy daily. In our previous study [8], each intervention period lasted only three days (in total, nine days), which was considered too short to capture adequate functional changes. Therefore, in the present study, we extended each intervention period to one week, totaling two weeks, to allow for a more reliable evaluation of the intervention effects while maintaining clinical feasibility.

Intervention protocol

**Figure 2:**
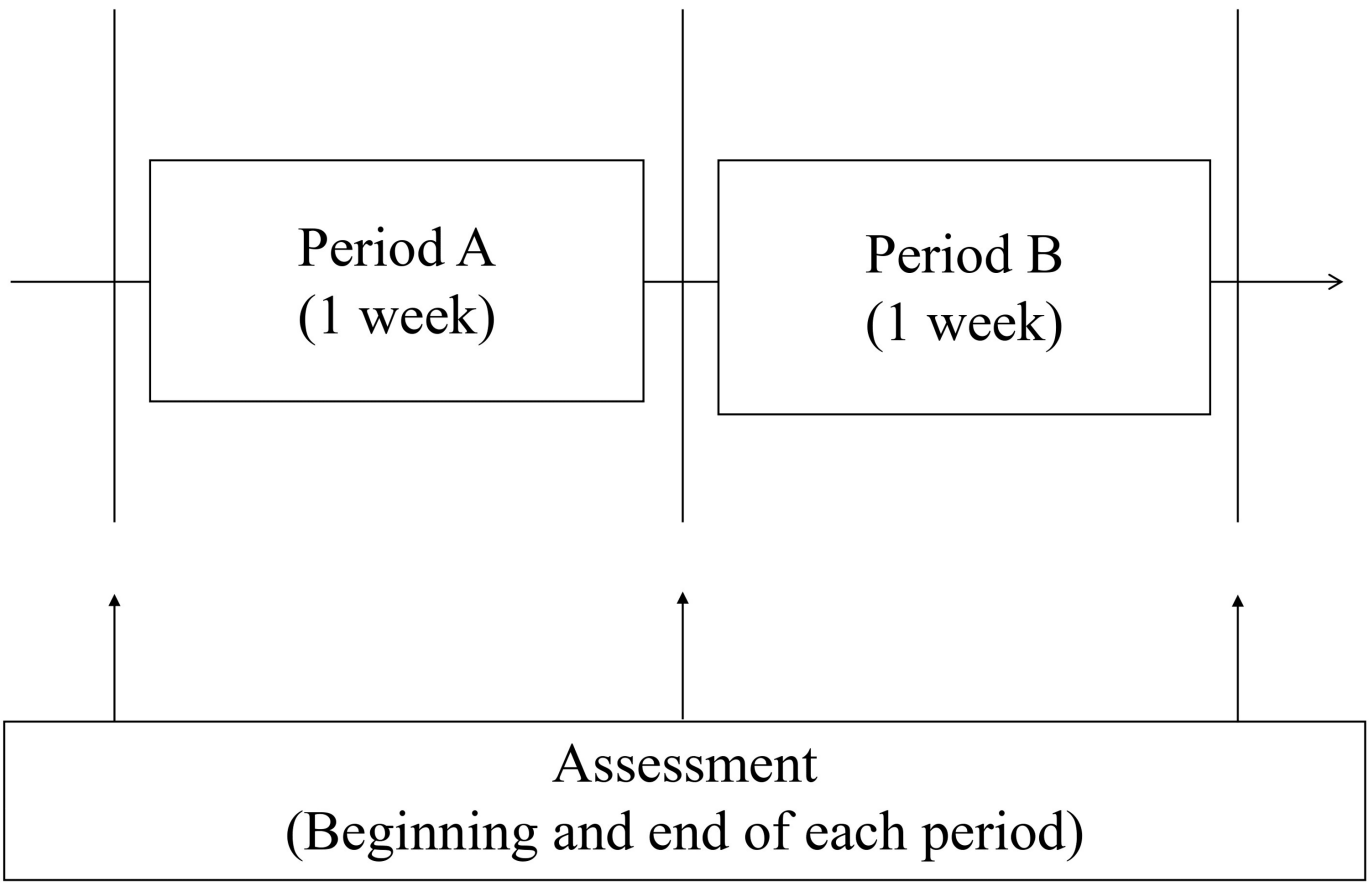
Intervention protocol. Assessments were conducted at the beginning and end of each period. The contents included the angle of the righting reaction, the distance movement of the center of pressure, the Trunk Impairment Scale, and the Functional Independence Measure. Conventional therapy (e.g., range-of-motion exercises, standing and sitting exercises, and walking exercises with a knee-ankle-foot orthosis (KAFO)) was performed for 20 minutes in both periods A and B. In period A, a KAFO was attached to the paralyzed side, and unstable board training was performed, in which the unstable board was adapted to the non-paralyzed side for 20 minutes. In period B, the same unstable board training as in period A was performed while applying transcutaneous electrical nerve stimulation to the sternocleidomastoid muscle on the affected side for 20 minutes.

The intervention protocol is shown in Figure 2. In period A, each physical therapy session lasted 40 minutes, consisting of 20 minutes of conventional physiotherapy and 20 minutes of unstable board training. Conventional physiotherapy included range-of-motion exercises, strengthening, and gait training with a KAFO attached to the paralyzed lower limb.

**Figure 3:**
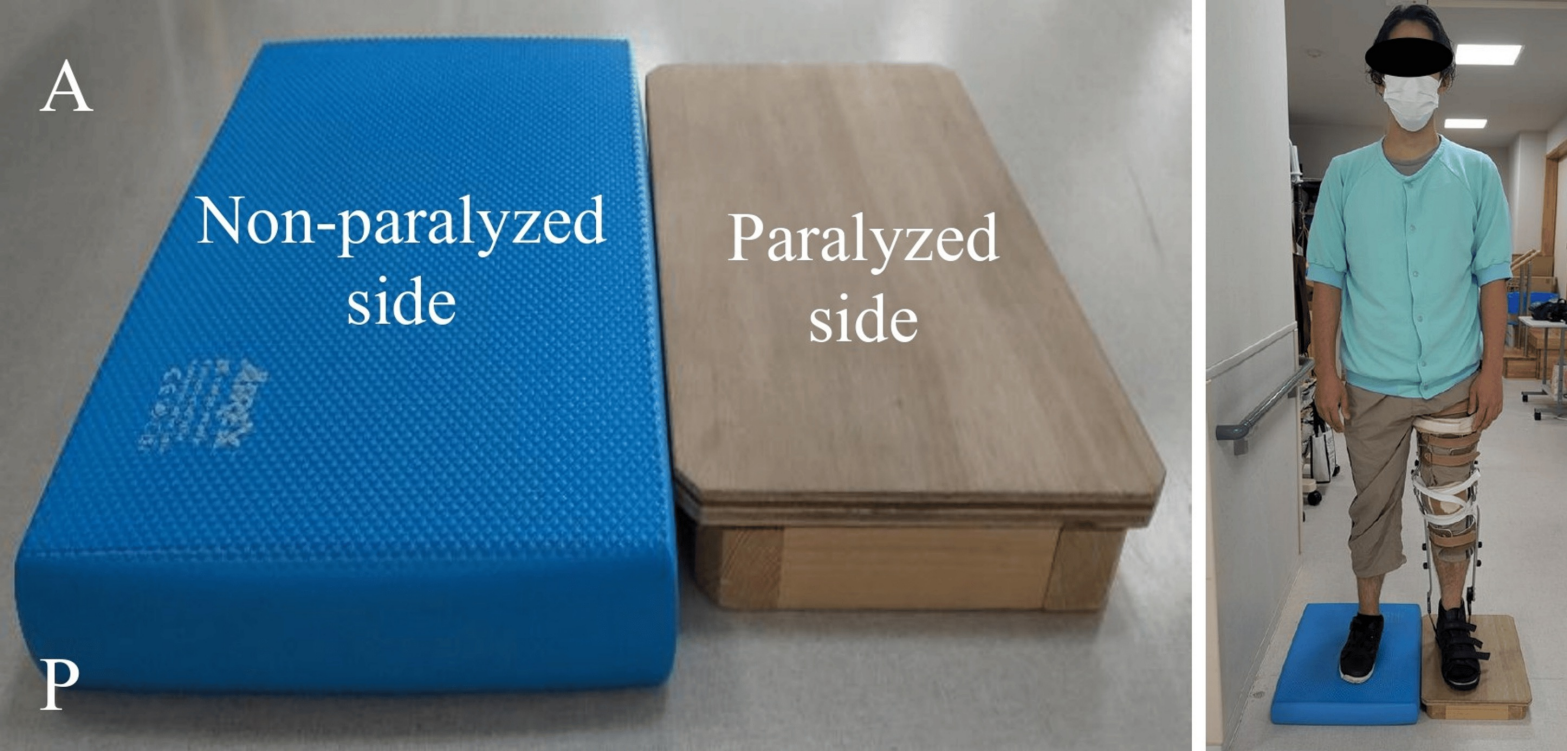
Settings for unstable board intervention (e.g., left hemiplegia). An unstable board (AIREX, Sanwa Trading Co., Ltd., Gifu, Japan) was placed under the non-paralyzed lower limb, while the paralyzed limb with a knee-ankle-foot orthosis (KAFO) rested on a stable platform of the same height. Training was performed in standing only and included weight-shifting and reaching tasks with the non-paralyzed upper limb in forward, lateral, and upward directions. Tasks were adjusted daily according to the patient's condition. No unstable board was used during sitting or gait training.

Unstable board training was performed only in the standing position and was based on a protocol described by Nagai et al. [8]. An unstable board (AIREX, Sanwa Trading Co., Ltd., Gifu, Japan) was placed under the non-paralyzed lower limb, whereas the paralyzed lower limb, equipped with a KAFO, was placed on a stable platform at the same height. During this training, participants performed weight-shifting tasks onto both lower limbs, as well as reaching exercises with the non-paralyzed upper limb in various directions (forwards, lateral, and upwards). The order and difficulty of tasks were adjusted daily according to the patient’s condition, with progression determined by task performance and tolerance. Exercises were performed under close supervision to ensure safety, with physical assistance provided to prevent falls. An unstable board was not used during sitting or gait training (Figure 3).

All interventions were primarily conducted by a single physical therapist. On days when the therapist was unavailable, another physical therapist provided the intervention following verbal and written handover instructions to ensure consistency.

**Figure 4:**
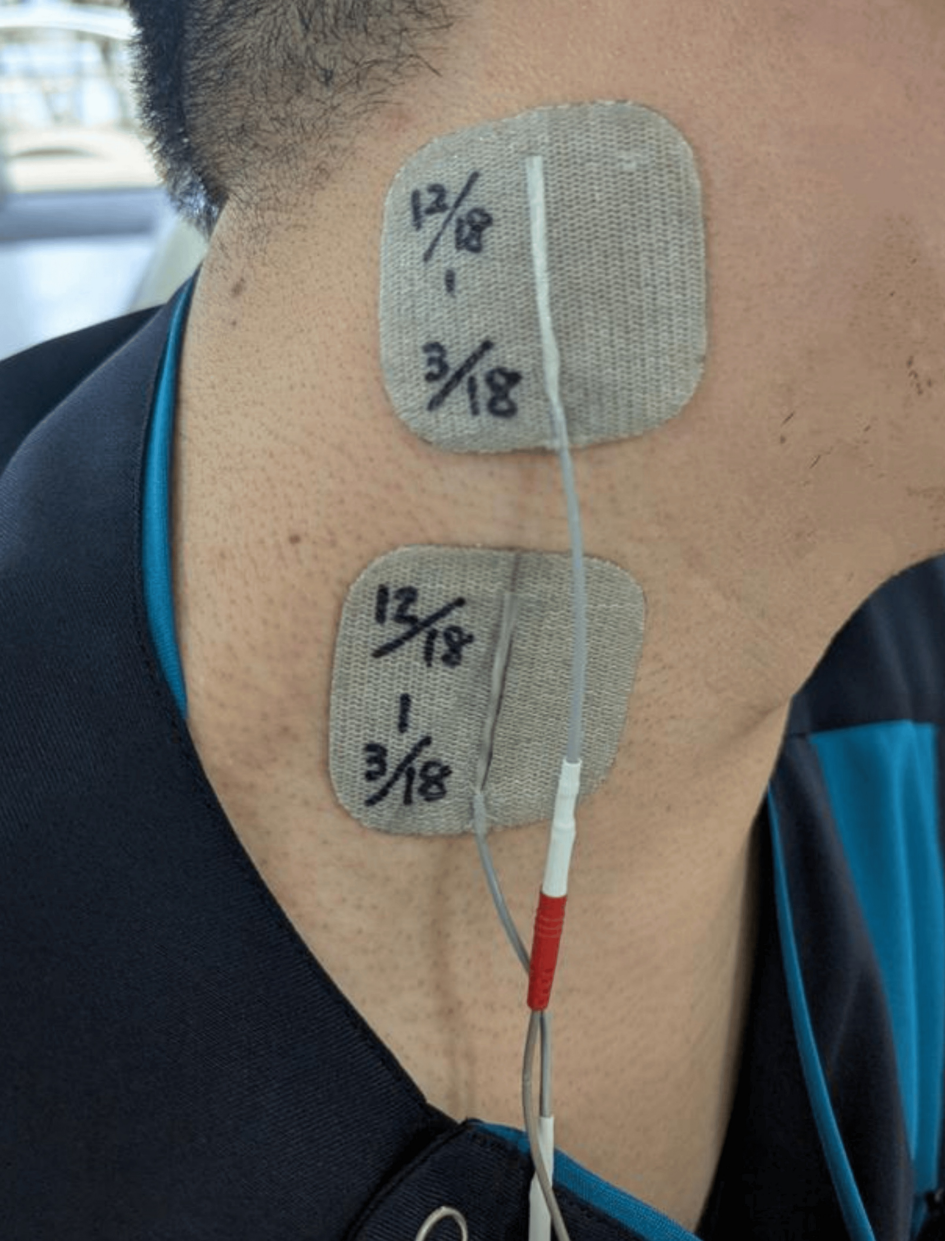
Electrode attachment position of transcutaneous electrical nerve stimulation (e.g., right hemiplegia). A transcutaneous electrical nerve stimulation (TENS) electrode was placed at the center of the sternocleidomastoid muscle. The TENS device (Espage, Ito Ultrashort Wave Co., Ltd, Saitama, Japan) was set to 100 Hz, with a pulse width of 200 µs and a total time of 20 minutes. The stimulation intensity was adjusted according to the sensory threshold.

The protocol in period B was the same as that in period A, with the addition of TENS applied to the paralyzed sternocleidomastoid muscle during a 20-minute unstable board training session. The TENS electrode was placed at the center of the sternocleidomastoid muscle (Figure 4). The TENS device (Espage, Ito Ultrashort Wave Co., Ltd, Saitama, Japan) was set to 100 Hz, with a pulse width of 200 µs and a total time of 20 minutes [18]. The stimulation intensity was adjusted according to the sensory threshold.

Assessment

Neurological and Neuropsychological Assessments

Motor function was assessed using the FMA and the BRS. Sensory function was assessed using the FMA protocol to evaluate superficial (light touch and pinprick) and deep (proprioception) sensations in the upper and lower limbs. Walking ability was assessed using the FAC, and cognitive function was evaluated using the MMSE. Trunk function was measured using the TIS, and ADLs were assessed using the FIM. These assessments were conducted by two physical therapists with three and five years of clinical experience. The TIS has shown good reliability and validity in stroke patient populations. Other assessment measures, such as the FMA and FIM, are established assessment measures widely used in stroke rehabilitation and are generally considered to have acceptable psychometric properties.

Lateral Sitting Balance

**Figure 5:**
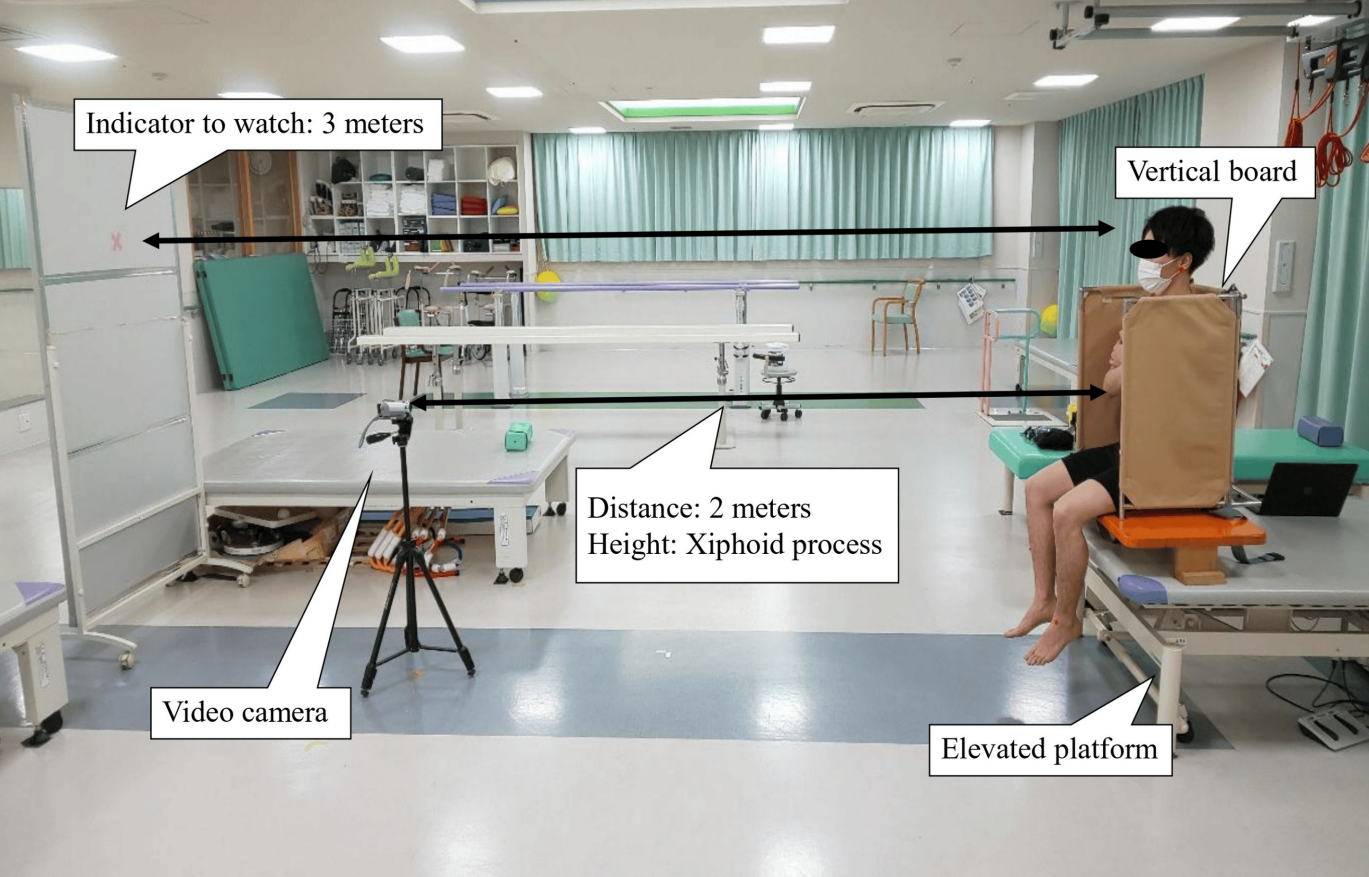
Environment setting for righting reaction assessment.

Lateral sitting balance was assessed using the RR angle and COP displacement. The RR angle was evaluated based on the method published by Nagai et al. [7]. The RR evaluation was conducted by two physiotherapists with three and eight years of clinical experience, who were blinded to the intervention. A VB was placed on an elevated platform, and a seat pressure distribution sensor (SR Soft Vision, Sumitomo Riko Co., Ltd., Aichi, Japan) was positioned on the VB. The system contained 256 sensors with a sampling frequency of 20 Hz. The participants sat in the VB with their feet off the ground and were instructed to tilt their bodies 10° in the frontal plane. The RR task involved sequential tilting to the non-paralyzed, paralyzed, paralyzed, and non-paralyzed sides. “Paralyzed side tilt” is defined as inclining the sitting board toward the paralyzed side. A video camera (Everio, JVC Kenwood, Kanagawa, Japan) with a sampling rate of 30 Hz was positioned 2 m away from the VB and aligned with the participant’s xiphoid process (Figure 5).

**Figure 6:**
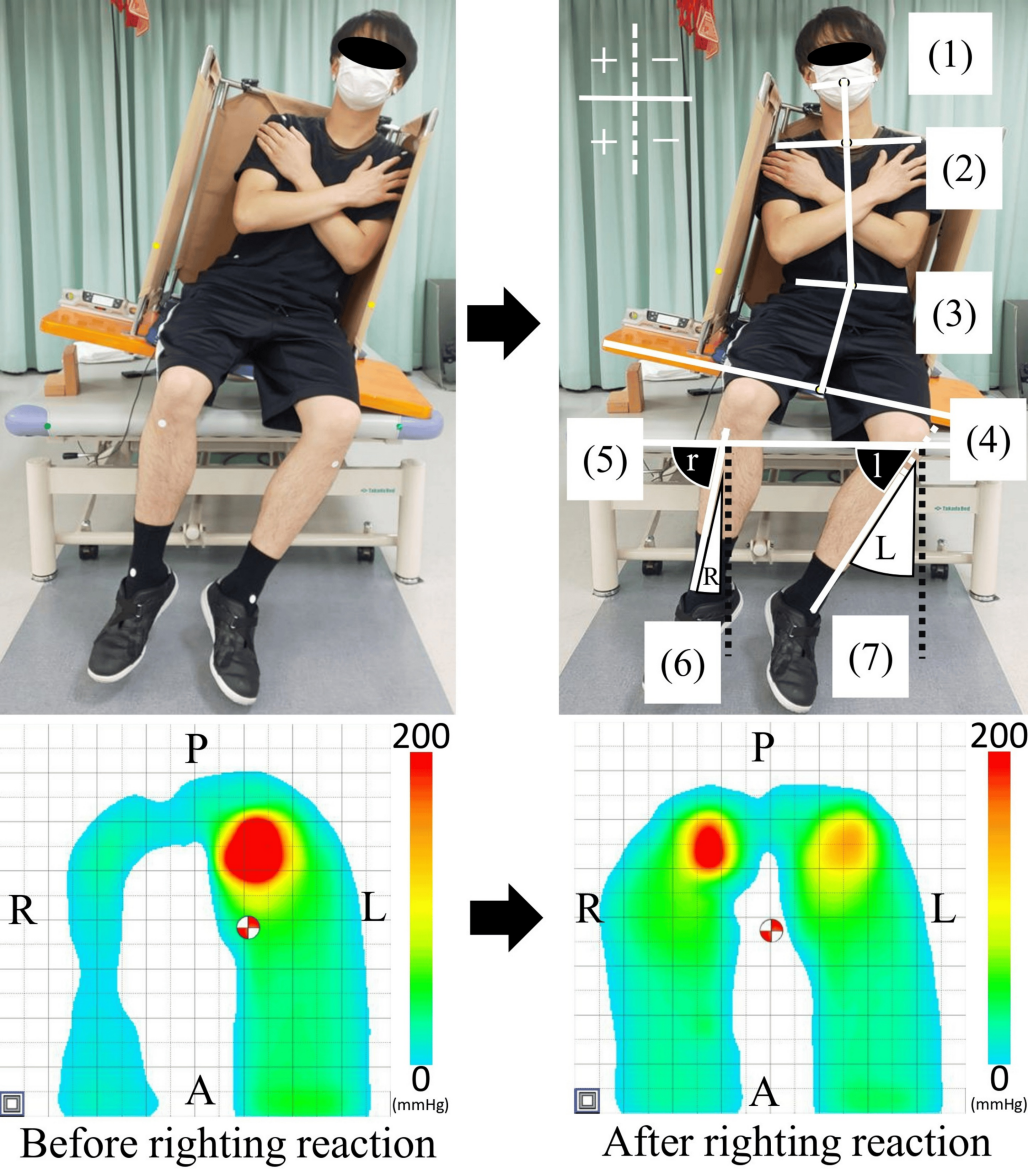
Calculation of the angle of the righting reaction and the center of pressure displacement distance (e.g., righting reaction toward the non-paralyzed side in left hemiplegia). Angle measurement (upper): (1) midpoint of the line connecting the two earlobes; (2) midpoint of the line connecting the two anterior acromions; (3) midpoint of the line connecting between anterior superior iliac spine on both sides; (4) midpoint of the upper edge of the vertical board; (5) line connecting the two frontal points of the elevated platform; (6) line connecting the right tibial tuberosity to the midpoint of the line connecting the right medial and lateral malleoli; (7) line connecting the left tibial tuberosity to the midpoint of the line connecting the left medial and lateral malleoli. Angle of the neck: between the line connecting (1), (2), and (3). Angle of the trunk: between the line connecting (2), (3), and (4). Angle of the right lower leg (R): 90 minus “r” (the angle between (5) and (6)). Angle of the left lower leg (L): 90 minus “l” (the angle between (5) and (7)). Positive and negative values were defined based on the reference dotted line on the image. Positive values indicated movement toward the non-paralyzed side and negative values indicated movement toward the paralyzed side. Center of pressure displacement distance (lower): red and white circles indicate the center of pressure. Number of sensors: 255; sampling frequency: 20 Hz; A: anterior; P: posterior; R: right; L: left.

Markers were placed at 12 anatomical locations, including the bilateral auricular lobules, anterior acromion, anterior superior iliac spines, tibial tuberosities, medial and lateral malleoli, and two reference points in front of the elevated platform. Video recordings were converted to still images using a free video to JPG converter (DVDVideoSoft Ltd., Roseau, Dominican Republic; https://www.dvdvideosoft.com/products/dvd/Free-Video-to-JPG-Converter.htm). Two images, one during the resting tilted sitting position and the other during RR, were analyzed using ImageJ (National Institutes of Health, Bethesda, MD) (Figure 6). The RR angle was calculated as the difference between the RR and resting tilted position, with the direction of the RR defined as positive.

The COP displacement during RR was calculated using data from the seat pressure distribution sensor (Figure 6). COP displacement was determined over one second during the resting tilted sitting position and over 10 seconds during the RR task.

Results

The results of the RR angle, COP displacement, TIS, and FIM for the two cases are summarized in Table 2. No adverse events, such as falls, were observed during the intervention period. Importantly, neither case involved shoulder contact with the VB during assessments, ensuring that the RR measurements were not affected by physical limitations.

**Table 2:** Results of the lateral sitting balance, trunk function, and activities of daily living. Intervention of A: Using an unstable board. Intervention of B: Using an unstable board and transcutaneous electrical nerve stimulation. RR, righting reaction; COP, center of pressure; TIS, Trunk Impairment Scale; FIM, Functional Independence Measure.

		Before Period A	After Period A	After Period B
The angle of RR toward the paralyzed side (°)				
Neck	Case 1	9.6	10.8	8.9
	Case 2	21.9	15.8	10.6
Trunk	Case 1	11.4	12.4	15.9
	Case 2	4.5	10.3	16.8
Paralyzed leg	Case 1	0.4	-1.2	-0.2
	Case 2	6.0	1.8	-1.8
Non-paralyzed leg	Case 1	8.0	4.3	1.6
	Case 2	7.7	8.9	3.3
The angle of RR toward the non-paralyzed side (°)				
Neck	Case 1	8.2	8.4	7.9
	Case 2	20.3	14.0	10.8
Trunk	Case 1	13.7	15.9	18.5
	Case 2	10.5	15.3	16.2
Paralyzed leg	Case 1	2.1	4.9	2.8
	Case 2	-1.1	-2.6	1.6
Non-paralyzed leg	Case 1	9.7	5.3	6.7
	Case 2	6.5	6.8	5.9
The movement distance of the COP of RR (mm)				
Toward the paralyzed side	Case 1	4.65	8.51	14.38
	Case 2	0.41	6.30	13.58
Toward the non-paralyzed side	Case 1	6.88	9.74	14.14
	Case 2	0.43	6.60	16.00
TIS (points)				
Static item	Case 1	7	7	7
	Case 2	4	4	7
Dynamic item	Case 1	4	4	4
	Case 2	0	0	4
Coordination item	Case 1	0	1	1
	Case 2	0	0	1
Total	Case 1	11	12	12
	Case 2	4	4	12
FIM (points)				
Mortar item	Case 1	32	32	34
	Case 2	26	26	33
Cognitive item	Case 1	11	11	11
	Case 2	14	14	14
Total	Case 1	43	43	45
	Case 2	40	40	47

RR Angles: Paralyzed Side

In case 1, the RR angle of the neck increased from 9.6° before period A to 10.8° after period A, but then decreased to 8.9° after period B. The trunk angle increased from 11.4° before period A to 12.4° after period A and further to 15.9° after period B.

In case 2, the RR angle of the neck markedly decreased from 21.9° before period A to 15.8° after period A and 10.6° after period B. In contrast, the trunk angle increased from 4.5° before period A to 10.3° after period A and 16.8° after period B.

RR Angles: Non-paralyzed Side

In case 1, the RR angle of the neck was 8.2° before period A, 8.4° after period A, and 7.9° after period B. The trunk angle increased from 13.7° before period A to 15.9° after period A and 18.5° after period B.

In case 2, the RR angle of the neck decreased from 20.3° before period A to 14.0° after period A and 10.8° after period B. The trunk angle improved from 10.5° before period A to 15.3° after period A and 16.2° after period B.

COP Movement Distance

In case 1, the COP displacement toward the paralyzed side steadily increased from 4.65 mm before period A to 8.51 mm after period A and 14.38 mm after period B. Similarly, for the non-paralyzed side, the distance increased from 6.88 mm before period A to 9.74 mm after period A and 14.14 mm after period B.

In case 2, the COP displacement toward the paralyzed side showed significant improvement, increasing from 0.41 mm before period A to 6.30 mm after period A and 13.58 mm after period B. For the non-paralyzed side, the distance increased from 0.43 mm before period A to 6.60 mm after period A and 16.00 mm after period B.

TIS Score

In case 1, the TIS score was 11 points before period A and improved to 12 points after period A. This score was maintained throughout period B. In case 2, the TIS score remained at four points before and after period A but showed a marked improvement to 12 points after period B.

FIM Score

In case 1, the motor item score of the FIM remained at 32 points before and after period A, but increased to 34 points after period B. The cognitive item score remained constant at 11 points throughout all periods, resulting in a total FIM score of 43 points before and after period A, which increased to 45 points after period B.

In case 2, the motor item score of the FIM remained at 26 points before and after period A, but improved to 33 points after period B. The cognitive item score remained stable at 14 points across all periods, leading to a total FIM score of 40 before and after period A, which increased to 47 after period B. These improvements were particularly evident in transfer and toileting items, totaling seven points (three points in transfer, two points in toileting, and one point each in bladder and bowel management) gained across these domains; these gains reflect a combination of rehabilitation and ADL practice with nursing assistance.

## Discussion

We are not aware of previous case reports that have examined the combination of unstable board training for the non-paralyzed lower limb with TENS applied to the paralyzed sternocleidomastoid muscle in stroke patients requiring substantial assistance with ADL. We hypothesized that this combined approach would be more effective in improving lateral sitting balance and trunk function than unstable board training alone. In our cases, adding neck TENS to unstable board training led to greater improvements in RR angles and COP displacement than training without TENS. These findings suggest that sensory augmentation through TENS may complement the proprioceptive and postural adaptation benefits of unstable board training.

Unstable board training has been reported to promote weight-shifting strategies and activate trunk muscles, contributing to improved postural control [11,12]. Sensorimotor training in an unstable environment enhances dynamic proprioceptive input and facilitates sensorimotor integration, thereby improving postural control [19]. Moreover, neuroimaging studies have demonstrated that dynamic balance training, performed as a whole-body balancing task on a movable platform, leads to structural and functional neuroplastic changes in cortical regions responsible for sensory and motor integration [13]. Such training increases grey matter volume in the parietal and frontal cortices and is associated with improved postural stability. Additionally, enhanced cortical activity in the frontocentral and centroparietal regions during unstable sitting suggests its effect on postural control [20]. However, owing to the risk of falls, its use has been largely limited to patients with mild hemiplegia [11]. To overcome this, our modified protocol stabilized the paralyzed limb with a KAFO, allowing safe dynamic sitting training for patients with more severe impairments [8].

TENS has been reported to modulate afferent sensory input and influence cortical and subcortical structures involved in postural control, including the insular cortex, primary somatosensory cortex (S1), and vestibular-related areas [14,15]. For example, Pérennou et al. [16] demonstrated that TENS applied to the neck improved postural stability in stroke patients with spatial neglect, suggesting its potential to facilitate somatosensory and vestibular integration through stimulation of the parieto-insular-vestibular network. While our study did not directly assess brain or muscle activity, the observed improvements may reflect these mechanisms as proposed in previous studies. However, the specific contribution of TENS to postural adaptation in the present study remains speculative and should be interpreted with caution.

Our findings suggest that TENS may enhance the effectiveness of unstable board training by increasing afferent sensory input, facilitating sensorimotor integration, and supporting motor adaptation mechanisms. Yada et al. [18] reported that task-related trunk training combined with sensory electrical stimulation improved trunk RR angles in stroke patients, indicating that sensory facilitation can promote motor adaptation. Our results extend this perspective by showing that adding TENS to the neck during unstable board training may yield greater improvements in sitting balance than unstable board training alone. Moreover, as trunk function is strongly associated with ADL performance [1], the improvement in trunk control and the TIS score observed in case 2 may contribute to better functional outcomes.

Previous studies have reported that TENS alone and unstable board training alone can have positive effects on post-stroke motor recovery. For example, TENS has been shown to improve balance by enhancing somatosensory input and modulating cortical excitability [14], while unstable board training has been associated with improvements in trunk function [8]. In our cases, the combined use of TENS and unstable board training appeared to have complementary effects, leading to improvements in both trunk control and postural stability. These findings suggest that incorporating sensory facilitation with dynamic balance training may provide additional benefits beyond those reported for each intervention alone.

Importantly, in interpreting these changes, we considered thresholds for the minimal clinically important difference (MCID) [8]. For RR toward the non-paralyzed side, MCID was 3.9° for the neck, 2.2° for the trunk, and 4.7 mm for COP displacement. For RR toward the paralyzed side, MCID was 3.6° for the neck, 3.1° for the trunk, and 3.3 mm for COP displacement. Improvements in case 2 exceeded several of these thresholds, suggesting that the observed gains were clinically meaningful. In contrast, improvements in case 1 were smaller and did not consistently surpass the MCID values, possibly due to more severe sensory impairments.

Notably, the two cases demonstrated different response patterns. Smaller improvements were observed in case 1, who was evaluated 116 days after stroke onset, than in case 2, who was evaluated after 62 days and had relatively preserved trunk function with a baseline TIS score of 11. This may reflect the establishment of compensatory postural strategies, which could have limited the additional benefit of sensory facilitation. In contrast, case 2 presented with markedly lower baseline trunk function (TIS score of 4), mild superficial and deep sensory deficits, and mild unilateral spatial neglect. Despite these impairments, case 2 exhibited substantial gains, particularly in the dynamic sitting balance and coordination subscales of the TIS, suggesting that the combined intervention may have specifically enhanced postural adaptability and trunk coordination. These inter-case differences highlight that baseline trunk function, sensory integrity, and associated cognitive-perceptual factors such as neglect may strongly influence responsiveness to interventions that combine proprioceptive and sensory augmentation.

Taken together, these findings support the hypothesis that combining dynamic proprioceptive input from unstable board training with sensory augmentation via TENS could offer a more comprehensive and effective approach to improving sitting balance in stroke survivors. Simultaneously, the variability between our two cases underscores the importance of considering sensory integrity when selecting candidates for such interventions. Future neurophysiological and functional studies are warranted to explore these mechanisms and identify which patient subgroups are most likely to benefit.

Limitations

This case report has several limitations. First, the use of an AB design limits the ability to fully verify the effectiveness of the intervention. While an ABA or ABAB design would have provided stronger evidence, such designs were ethically and practically difficult to implement in this population. In addition, the use of an alternating AB design without a washout period raises the possibility that the effects observed in period B were influenced by carryover from period A. Furthermore, although we extended the intervention period to two weeks based on our previous study [5], in which a nine-day protocol was considered too short to capture functional changes, the relatively short duration still limits conclusions about the long-term sustainability of the observed effects. Second, no neurophysiological measures (e.g., brain imaging or muscle activity) were used, limiting the ability to directly examine the mechanisms underlying the observed improvements. Third, while improvements were noted following the addition of TENS, it remains unclear whether these effects were due to TENS alone or to the synergistic combination with unstable board training. Fourth, we did not systematically collect data on the patients’ chief complaints or perform structured follow-up evaluations after the intervention period. Therefore, the relationship between subjective improvements and objective outcomes, as well as the persistence of the effects, could not be determined. Fifth, although no shoulder or trunk contact with the vertical board was observed in either of the present cases, such contact could potentially occur in patients with larger body builds. This might effectively widen the base of support and influence the measurement of RR, representing a methodological limitation to be considered in future applications. Sixth, the generalizability of these findings is limited by the small sample size and the case report design. Further research with larger cohorts and objective neurophysiological assessments is necessary to validate these results and clarify the mechanisms involved. Finally, the Montreal Cognitive Assessment (MoCA) is highly sensitive in detecting cognitive impairment after stroke, but it was not used in this study. At the time of data collection, our team did not have the official certification required for MoCA use. Future studies should incorporate the MoCA or other validated neuropsychological assessment tools to more accurately assess cognitive function after stroke.

## Conclusions

This case report demonstrated that combining unstable board training with TENS applied to the neck was associated with greater improvements in lateral sitting balance than unstable board training alone in two stroke patients who required substantial assistance with ADLs. These results suggest that TENS may enhance afferent sensory input and facilitate sensorimotor integration, potentially supporting more effective postural adaptation. Future studies with larger sample sizes and objective neurophysiological measures are warranted to further validate these findings and explore their applicability to broader stroke populations.
